# Stable and Accurate Estimation of SOC Using eXogenous Kalman Filter for Lithium-Ion Batteries

**DOI:** 10.3390/s23010467

**Published:** 2023-01-01

**Authors:** Qizhe Lin, Xiaoqi Li, Bicheng Tu, Junwei Cao, Ming Zhang, Jiawei Xiang

**Affiliations:** 1 College of Mechanical and Electrical Engineering, Wenzhou University, Wenzhou 325035, China; 2 Ebara Great Pumps Co., Ltd., Wenzhou 325200, China

**Keywords:** lithium-ion battery, SOC, second-order RC equivalent model, eXogenous Kalman filter, estimation

## Abstract

The state of charge (SOC) for a lithium-ion battery is a key index closely related to battery performance and safety with respect to the power supply system of electric vehicles. The Kalman filter (KF) or extended KF (EKF) is normally employed to estimate SOC in association with the relatively simple and fast second-order resistor-capacitor (RC) equivalent circuit model for SOC estimations. To improve the stability of SOC estimation, a two-stage method is developed by combining the second-order RC equivalent circuit model and the eXogenous Kalman filter (XKF) to estimate the SOC of a lithium-ion battery. First, approximate SOC estimation values are observed with relatively poor accuracy by a stable observer without considering parameter uncertainty. Second, the poor accuracy SOC results are further fed into XKF to obtain relative stable and accurate SOC estimation values. Experiments demonstrate that the SOC estimation results of the present method are superior to those of the commonly used EKF method. It is expected that the present two-stage XKF method will be useful for the stable and accurate estimation of SOC in the power supply system of electric vehicles.

## 1. Introduction

State of Charge (SOC) is the key index closely related to lithium-ion battery performance and safety to reflect the characteristics the power supply system of electric vehicles. Online and high precision estimate SOC is required by a battery management system to prevent battery overcharge and discharge, low power current limiting threshold, vehicle control strategy threshold, etc [1]. Generally, SOC cannot be measured directly; hence, it is usually estimate by detecting terminal voltage, current, impedance, temperature, and other parameters [2,3,4]. The indirect measurement of these physical quantities will lead to the high nonlinearity of SOC estimations. Therefore, online and effective control SOC can improve and prolong the efficiency and cycle life of lithium-ion batteries. Hence, it is of great significance to estimate the SOC of lithium-ion batteries. SOC estimation method based on parameter identification, such as neural networks [5,6], the Kalman filter (KF) method, the extended Kalman filter (EKF) method [7], and the unscented Kalman filter method [8].

Artificial intelligence (AI) models can automatically learn features without extensive prior knowledge and have been widely used for unknown abnormal condition detection and prediction [9,10,11], especially for SOC estimation for lithium-ion batteries [12,13,14]. For example, an adaptive back propagation neural network was introduced to improve the SOC estimation accuracy of an unscented Kalman filtering algorithm [15]. A recurrent convolutional neural network (RCNN) was employed for SOC prediction of lithium-ion batteries [16], and a stacked encoder–decoder bi-directional long short-term memory (LSTM) was used to estimate SOC for the electric vehicle and hybrid electric vehicle [17]. Generally, the key factor for the success of AI model application is sufficient or even complete training samples for obtaining a high classification accuracies in real-world applications. However, there is no easy way to acquire numerous training samples from running electric vehicles under complex working conditions in engineering practice. Consequently, the Kalman filter technique and its improved versions are the best choice for SOC estimation.

The theoretical basis of linear KF is well established for stochastic processes, estimation, and control [18]. Furthermore, the combination of the adaptive KF method and the battery Thevenin equivalent circuit are dynamically combined to estimate the SOC of an electric vehicle power battery with measurement noises [19]; Robust KF was presented for estimating the non-Gaussian probability density model problem [20] by using the expectation maximization (EM) algorithm [21].Generally, EKF is a kind of relatively broad estimation method using nonlinear KF approximation lacking global stability, essentially caused by a feedback loop introduced when calculating the approximation (linearization) of a local linear model. The key is to linearize the current state estimate, and the linearization will be poor due to the poor initialization of the state estimate. The KF update may not reduce the estimate error and thus prevent the error from converging [22]. Recently, researchers have provided many improvements to the KF method. Liu [23] proposed an adaptive square root unscented Kalman filter (ASRUKF) method to estimate the SOC of lithium-ion batteries, and the effectiveness of the ASRUKF method has been verified through experiments under different operating conditions with better accuracy, robustness and convergence. To overcome the regression least squares algorithm-based extended Kalman filter (RLS-EKF), an adaptive forgetting factor-based RLS-EKF (AFFRLS-EKF) SOC estimation strategy was used to improve the accuracy of SOC estimation under changes in battery charge and discharge conditions [24].

In summary, the above research focuses on finding the relative best preprocessing method to improve the combination of improvement of KF and the second-order RC equivalent model to guarantee stability for SOC estimations. However, the structural parameters of a stochastic resonance system have a great impact on its output; each input signal will correspond to a set of optimal structural parameters. Therefore, another way is to deal directly with the linear Kalman filter (LKF). To enhance the performance of LKF, a two-stage estimation strategy called the eXogenous Kalman filter (XKF) is developed by Johansen and Fossen [25]. The model linearization is accomplished by an auxiliary state estimate to the LFK, which does not depend on the LKF’s own state estimation. Therefore, the stability properties of XKF are inherent in the auxiliary state estimator [26]. Hence, XKF was applied to generate a globally exponentially stable observer for the visualization and motion prediction of ships to solve the problem of kinematic nonlinearities [27]. Chen et al. developed an observer-based two-stage extended Kalman filter (TSEKF) for a satellite attitude control system (ACS) with unknown time-varying actuator faults [28]. Ma et al. proposed a modified XKF (MXKF) for fault-tolerant stability control of a three-phase permanent magnet synchronous motor control system with encoder faults [29].

To perform KF and/or EKF and their improved versions, a second-order RC equivalent model of the lithium-ion battery is commonly employed to serve as a baseline model with changeable parameters [30,31]. Xia et al. systematically proposed a hybrid of the second-order RC equivalent circuit and parameters of the battery model were determined by the forgetting factor least-squares method, the state space equation observer method, and the adaptive extended Kalman particle filter (AEKPF) using a particle filter (PF) and adaptive Kalman filter (AKF) [32,33,34]. Guo et al. developed a combination of the second-order RC equivalent circuit and parameters and the parameter estimator using the least squares method with a forgetting factor and the adaptive UKF algorithm to jointly estimate SOC [35]. Falai et al. applied the second-order RC equivalent circuit model to predict the SOC of on-road lithium-ion battery tests and further validated the global battery-to-wheels efficiency [36].

These works paved the way towards stability and accurate prediction of the SOC of lithium-ion batteries for the power supply system of electric vehicles. However, the combination of KF or EKF and the second-order RC equivalent circuit model will lead to accurate SOC estimation but cannot keep its stability. Moreover, XKF is in nature a stability estimator suitable for SOC estimation. In this paper, a combination method using the second-order RC equivalent circuit model and XKF is developed to estimate the SOC of a lithium-ion battery. For this purpose, this paper is structured as follows: equivalent circuit model for a lithium-ion battery is given in Section 2. In Section 3, using the equivalent circuit model of lithium-ion batteries, approximate SOC estimation values are observed with relatively poor accuracy by a stable observer without considering parameter uncertainty. The approximate SOC results serve as the pre-treated data to activate XKF to obtain the relatively stable and accurate SOC estimation values in Section 4. Finally, conclusion remarks are given in Section 5.

## 2. Equivalent Circuit Model for a Lithium-Ion Battery

The equal-effect circuit model makes the relationship between voltage and current of the battery in practice simulated by the common basic circuit components, such as resistance, capacitance, and voltage source. A large number of investigations have shown that there is a certain relationship between the residual power and the parameters of lithium batteries and the easy-access parameters. Therefore, equivalent circuit models, such as the Rint model [37], the Randles model [38],and the *n*th-order RC model [39], have been developed to understand the relationship between parameters and SOC of lithium batteries. Moreover, equivalent circuit models can also be employed to simulate the characteristics of lithium-ion batteries for power supply system in electric vehicles. Generally, the second-order RC equivalent circuit model is a commonly used equivalent circuit model for serving as a baseline to predict SOC of lithium-ion batteries [30,31,32,33,34,35,36].

**Figure 1:**
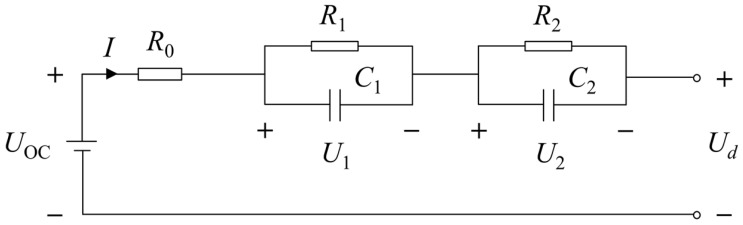
The second-order RC equivalent circuit model.

The second-order RC equivalent circuit model with a simple circuit structure can well reflect the dynamic and static characteristics of the lithium-ion battery in the power supply system of electric vehicles. Therefore, it has good real-time performance to meet the requirements for the development of battery management systems. Figure 1 shows the second-order RC equivalent circuit model, which consists of battery electromotive force *Uoc*, an ohmic internal resistance of battery *R*_0_, and a series connection of two parallel capacitive resistance circuits. In Figure 1, *U_d_* is the open circuit voltage, *R*_1_ and *R*_2_, *C*_1_ and *C*_2_ are the polarization internal resistances and the polarization capacitors of the battery, respectively, with the voltage *U*_1_ and *U*_2_. It is worth pointing out that *C_1_* and *C_2_* are given to simulate the dynamic characteristics of the voltage gradient in the process of battery polarization.

According to the second-order RC equivalent circuit model shown in Figure 1, the following equations can be obtained as:

(1) $$\{\begin{array}{c} U_{oc}=ƒ\left(SOC\right)\\ SOC=SOC_{0}-\frac{1}{Q_{N}}\int_{0}^{T}\eta Idt\\ \begin{array}{c} C_{1}\cdot \frac{dU_{1}}{dt}+\frac{U_{1}}{R_{1}}=I\\ C_{2}\cdot \frac{dU_{2}}{dt}+\frac{U_{2}}{R_{2}}=I\\ \frac{U_{0}}{R_{0}}=I \end{array} \end{array}$$

where $SOC_{0}$ and $Q_{N}$ are the initial value of SOC, and the rated capacity of the battery, respectively. η is Coulomb efficiency can be obtained by charging and discharging experiments of a battery, generally can be set to 1, and *T* is the period of charging and discharging cycle of the battery.

It is worth pointing out that in the present investigation, only the *U_1_* and *U_2_* are changed with time. Therefore, the state-space equation of Equation (1) can be written as:

(2) $$\left[\begin{array}{c} SOC\\ \dot{U_{1}}\\ \dot{U_{2}} \end{array}\right]=\left[\begin{array}{ccc} 1 & 0 & 0\\ 0 & 1-\frac{1}{R_{1}C_{1}} & 0\\ 0 & 0 & 1-\frac{1}{R_{2}C_{2}} \end{array}\right]\cdot \left[\begin{array}{c} SOC_{0}\\ U_{1}\\ U_{2} \end{array}\right]+\left[\begin{array}{c} -\frac{\eta \cdot T}{Q_{N}}\\ \frac{1}{C_{1}}\\ \frac{1}{C_{2}} \end{array}\right]\cdot I\;$$

and

(3) $$U_{d}=U_{oc}\left(SOC\right)-U_{1}-U_{2}-I\cdot R_{0}$$

The state-space equation is discretized, and the discrete model of the equation can be obtained as:

(4) $$\left[\begin{array}{c} SOC_{k+1}\\ U_{1,k+1}\\ U_{2,k+1} \end{array}\right]=\left[\begin{array}{ccc} 1 & 0 & 0\\ 0 & 1-\frac{1}{R_{1}C_{1}} & 0\\ 0 & 0 & 1-\frac{1}{R_{2}C_{2}} \end{array}\right]\cdot \left[\begin{array}{c} SOC_{k}\\ U_{1,k}\\ U_{2,k} \end{array}\right]+\left[\begin{array}{c} -\frac{\eta \cdot T}{Q_{N}}\\ \frac{1}{C_{1}}\\ \frac{1}{C_{2}} \end{array}\right]\cdot I_{k}$$

(5) $$U_{d,k}=U_{oc}\left(SOC_{k}\right)-U_{1,k}-U_{2,k}-I_{k}\cdot R_{0}$$

## 3. Approximate SOC Estimation Using the Equivalent Circuit Model of Lithium-Ion Battery by Experimental Tests

**Figure 2:**
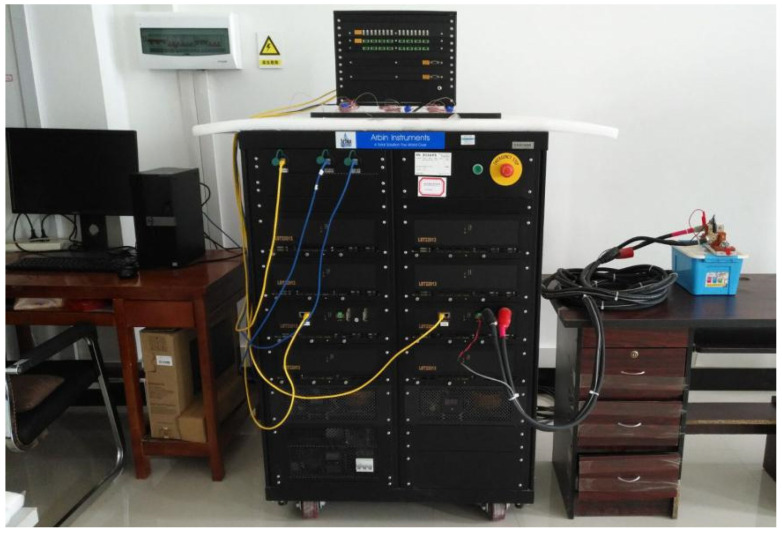
Arbin test rig and NCM532 lithium-ion batteries.

In the present study, the experiment test rig and test object are Arbin BT-ML-100V100A (Arbin Company) and NCM532 lithium-ion batteries (4.2 V, 24 Ah), respectively, as shown in Figure 2. The operating temperature range of the battery is −20 degrees Celsius ~50 degrees Celsius. In the experiments, the lithium-ion battery is tested with 1C pulse discharge, and the sampling time was 1 s/ time, the experimental temperature is under room temperature around 25 degrees Celsius. More details about how to use the Arbin BT-ML-100V100A test rig can be seen in the user’s manual of Arbin Company.

Electromotive force *Uoc* is the potential difference between the positive and negative poles when the battery is in a stable state. However, the direct measurement of *Uoc* is difficult, but it can be represented by the open-circuit voltage *U_d_* for *SOC* calculations.

Table 1 shows the relationship between *U_d_* and *SOC* during the test. Using polynomial curve fitting technique, the specific function relation between *U_d_* and *SOC* is:

(6) $$U_{\mathrm{oc}}=-8{.261\mathrm{SOC}}^{7}+0{.003158\mathrm{SOC}}^{6}-0{.04947\mathrm{SOC}}^{5}+0{.4086\mathrm{SOC}}^{4}-\;1{.898\mathrm{SOC}}^{3}+4{.873\mathrm{SOC}}^{2}-6.122\mathrm{SOC}+6.141$$

**Figure 3:**
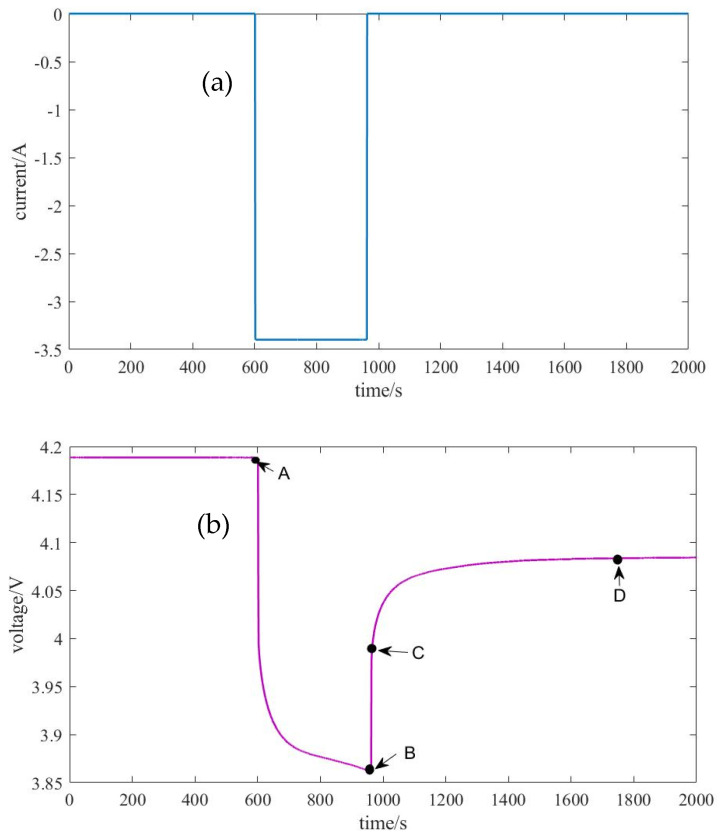
Single-stage discharge current and voltage curves: (**a**) the single-stage discharge current curve; (**b**) the single-stage discharge voltage curve. Notes: A and B are the starting and ending points of circuit in a discharge stage, respectively; C and D are the inflexion and end points of circuit in a quiescent stage.

Rebound voltage is the value of electrode potential deviation from equilibrium potential caused by equivalent impedance in the charging and discharging process of the battery, which mainly includes ohmic internal resistance and polarization internal resistance. The analysis of battery rebound voltage characteristics can provide a basis for the estimation of basic model parameters. Figure 3 shows the single-stage discharge current and voltage. As shown in segments A-B in Figure 3b, the voltage is dropped sharply at the beginning of discharge and then slowly decreased to point B. At the end of discharge, as shown in segments B-C-D, the voltage is rapidly raised and then increased slowly.

The segments of B-C-D in the voltage curve are selected for analysis. The voltage drop in segment B-C is caused by ohmic internal resistance $R_{0}$ as:

(7) $$R_{0}=\frac{U\left(C\right)-U\left(B\right)}{I}\;$$

where U(B) and U(C) are circuit voltages at point B and C.

For segment C-D, the zero-input voltage response is U(t)=U(D)·e−tτ, where U(D) is the circuit voltage at point D, τ = *RC* is the time constant of the *RC* of the series connection of two parallel capacitive resistance circuits as shown in Figure 1. The single stage discharge voltage curve shown in Figure 3b presents the shape of an exponential function. Therefore, the zero-input voltage response $U{\left(t\right)}_{1}$ and $U{\left(t\right)}_{2}$ of R_1_C_1_ and R_2_C_2_ circuits are:

U(t)1=U(D)1·e−tτ1

U(t)2=U(D)2·e−tτ2

where $\tau_{1}=R_{1}C_{1}$ and $\tau_{2}=R_{2}C_{2}$ with the restraint $\tau_{1}>\tau_{2}$ are the time constants of *R*_1_*C*_1_ and *R*_2_*C*_2_ circuits, respectively. Therefore, the terminal voltage at any time in segment C-D can be expressed as:

(8) UB(t)=Uocv(D)−U1(t)−U2(t)=Uocv(D)−IR1·e−tτ1−IR2·e−tτ2 

where $U_{ocv}\left(D\right)$ is the open-circuit voltage at point D.

Furthermore, Equation (8) can be written as follows:

(9) UB(t)=k0−k1·e−tτ1−k2·e−tτ2 

in which

(10) $$\{\begin{array}{c} \begin{array}{c} R_{1}=\frac{k_{1}}{I}\\ R_{2}=\frac{k_{2}}{I} \end{array}\\ C_{1}=\frac{I\cdot \tau_{1}}{k_{1}}\\ C_{2}=\frac{I\cdot \tau_{2}}{k_{2}} \end{array}$$

The voltage response data of the segment C-D is imported, and the function can be fitted and values of parameters$\;k_{0}$,$\;k_{1}$ and $k_{2}$ can be obtained. The parameters of the model can be obtained from (5) and (6)

Finally, the parameters of the second-order RC equivalent circuit model are identified and listed in Table 2.

**Figure 4:**
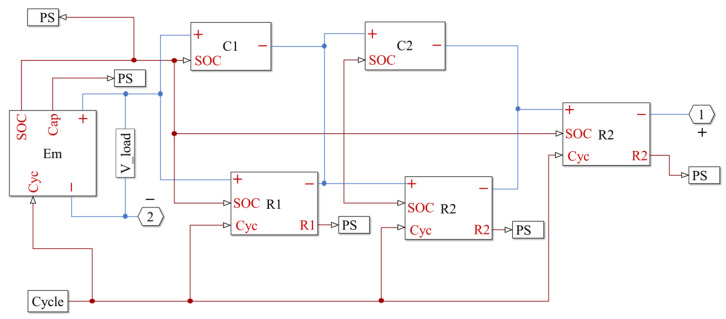
Block diagram of Simulink for the second-order RC equivalent circuit model.

**Figure 5:**
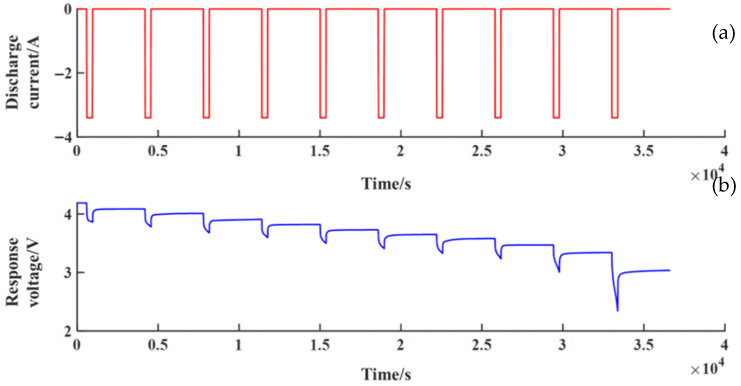
Response voltage curve from the Simulink simulation model: (**a**) the discharge current curve; (**b**) the response voltage curve.

**Figure 6:**
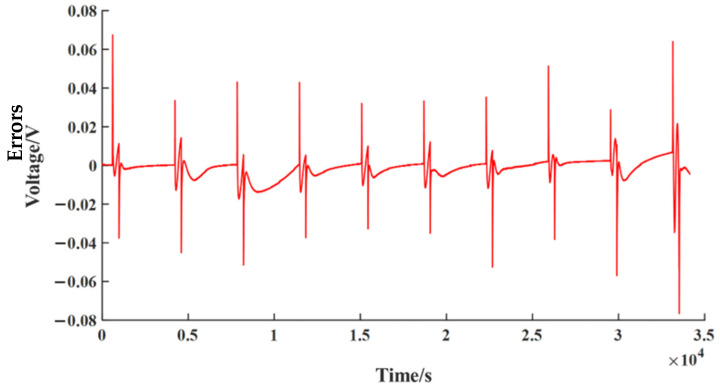
Error curve of response voltages between the Simulink simulations and experiments.

To validity the performance of the second-order RC equivalent circuit model, simulations using MATLAB/Simulink are given as shown in Figure 4. The NCM532 lithium-ion battery is tested using the Arbin BT-ML-100V100A test rig, and the measured values of 1C pulse discharge are used for the verification of the second-order RC equivalent circuit model. Comparing the measured values with simulated values through the second-order RC equivalent circuit model, the response voltage curve obtained from the Simulink simulation model is shown in Figure 5. Figure 6 shows the error curve of the response voltages between the Simulink simulations and the experiments. From Figure 6, we can clearly see that the errors in response voltages between the simulations and experiments are all within 7%. Therefore, the second-order RC equivalent circuit model we adopted herein can be employed to simulate the chemical reaction characteristics inside the battery with certain precision.

## 4. eXogenous Kalman Filter to Estimate SOC

### 4.1. Extended Kalman Filter to Estimate SOC

**Figure 7:**
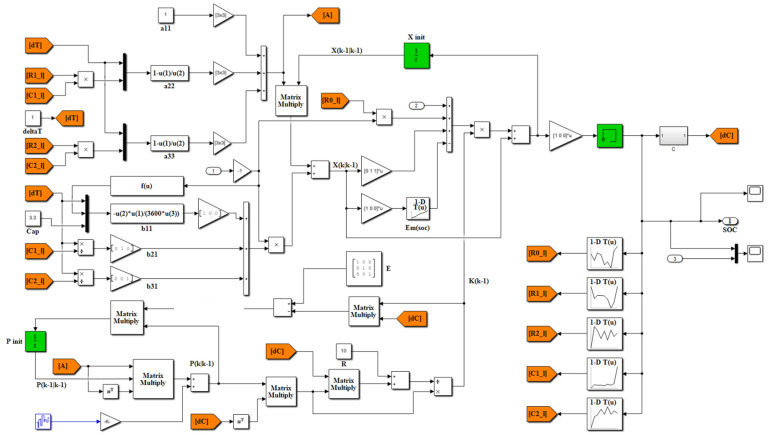
Block diagram of Simulink for the SOC estimation of the lithium-ion battery using EKF.

**Figure 8:**
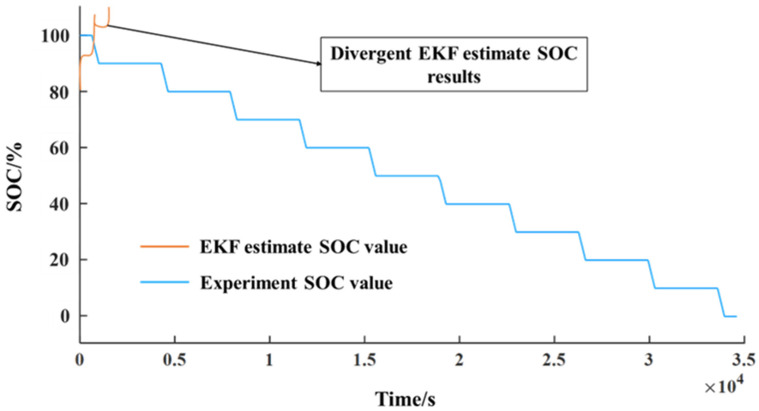
Divergent SOC estimation results using EKF.

KF uses the measured data of the state-space equation and output equation of the system to get the state or parameter to be estimated under the condition that the system noise, mathematical model, and initial state value are known. The standard Kalman filter can estimate the state variables of linear systems optimally and is a globally exponentially stable and robust algorithm. However, the lithium-ion battery itself is a nonlinear dynamic system, and the nonlinear system requires some extension or modification of KF, such as EKF, untraced KF, etc. More details about EKF can be seen in Ref. [7]. Using the second-order RC equivalent circuit model, the block diagram of EKF to estimate SOC by MATLAB/Simulink are given as shown in Figure 7. The divergent SOC estimation results by EKF are shown in Figure 8.

The core idea of the EKF algorithm is to expand nonlinear functions in nonlinear systems according to the first-order Taylor series, ignore second-order and higher-order terms, transform the original system into an approximate linearized model, and then use a linear Kalman filter algorithm for state estimation. In the process of estimating the SOC of lithium-ion batteries using EKF, as shown in Figure 8, the results are not convergent. The reason is that the feedback loop is introduced in the calculation of local linear model approximation (linearization), but the performance is determined by the quality of the state estimation initialization. However, generally, EKF cannot reduce estimation errors and thus prevent error convergence.

### 4.2. eXogenous Kalman Filter to Stablilty Estimate SOC

As mentioned in Section 4.1, if the quality of the state estimation initialization is poor, EKF will fail to estimate the SOC of the lithium-ion battery. Johansen and Fossen [25] developed the XKF and proved that XKF is a cascade of auxiliary state estimators not dependent on the linear Kalman filter’s own state estimation. The design of the auxiliary state estimator is listed below.

**Figure 9:**
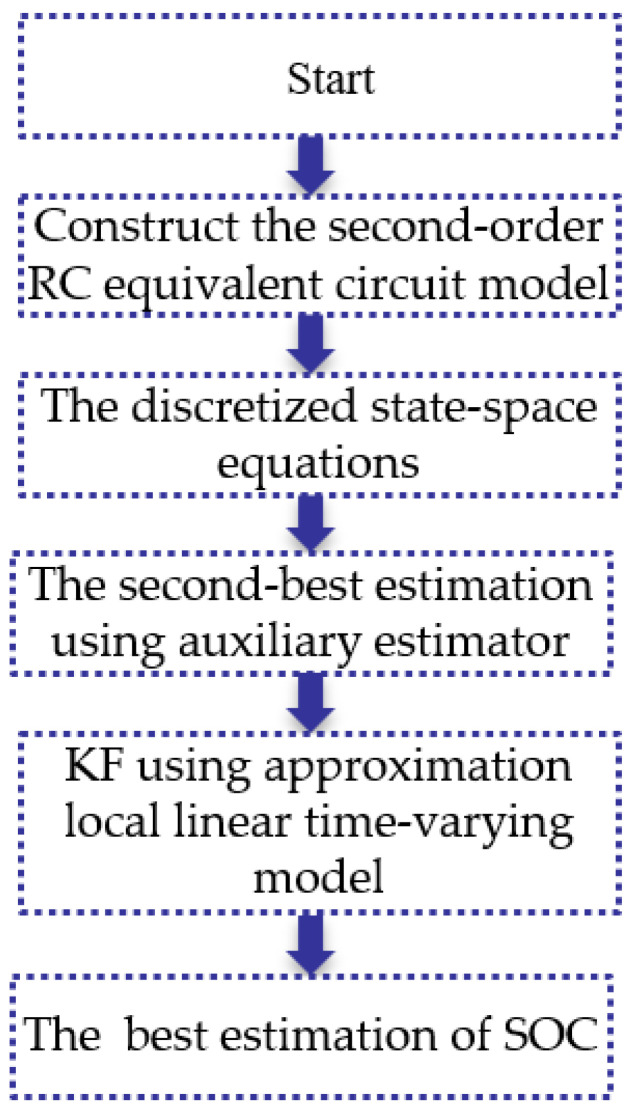
The flowchart of the SOC estimation using XKF.

The main requirement of an auxiliary state estimator is strong (nominal) stability in the absence of noise, preferably global exponential stability. In an XKF environment, its response to noise is considered less important. Therefore, the discretized state-space equations, as shown in Equations (4) and (5), are represented by:

(11) $$\left[\begin{array}{c} SOC_{k+1}\\ U_{1,k+1}\\ U_{2,k+1} \end{array}\right]=\left[\begin{array}{ccc} 1 & 0 & 0\\ 0 & 1-\frac{1}{R_{1}C_{1}} & 0\\ 0 & 0 & 1-\frac{1}{R_{2}C_{2}} \end{array}\right]\cdot \left[\begin{array}{c} SOC_{k}\\ U_{1,k}\\ U_{2,k} \end{array}\right]+\left[\begin{array}{c} -\frac{\eta \cdot T}{Q_{N}}\\ \frac{1}{C_{1}}\\ \frac{1}{C_{2}} \end{array}\right]\cdot I_{k}+w_{k}$$

(12) $$U_{d,k}=U_{oc}\left(SOC_{k}\right)-U_{1,k}-U_{2,k}-I_{k}\cdot R_{0}+v_{k}$$

in which, $w_{k}$ and $v_{k}$ are the uncorrelated and zero mean Gaussian white noises. Figure 9 shows the flowchart of the SOC estimation using XKF.

From Figure 9, we can see clearly that the differences of the XKF are: (1) the second-best estimation using auxiliary estimator; (2) KF using approximation local linear time-varying model to obtain the best estimation of SOC.

The main step of the auxiliary estimator is to calculate the rough ${\hat{U}}_{1}$ and ${\hat{U}}_{2}$ by Equation (13). By plugging into the lithium-ion battery’s equation of state, we can get the rough SOC estimate, $\hat{SOC}$. $x_{k}={\left[SOC_{k}U_{1,k}\;U_{2,k}\right]}^{T}$, where T denotes transpose.

Suppose$\;x_{k}={\left[{\hat{SOC}}_{k},\;{\hat{U}}_{1,k},\;{\hat{U}}_{2,k}\right]}^{T}$ the initial values ${\hat{U}}_{1,0}={\hat{U}}_{2,0}$=0, according to Equations (11) and (12), the second-best estimation values ${\hat{U}}_{1}$, ${\hat{U}}_{2}$ and $\hat{SOC}$ can be estimated using the state-space equations as:

(13) $$\left[\begin{array}{c} {\hat{SOC}}_{k+1}\\ {\hat{U}}_{1,k+1}\\ {\hat{U}}_{2,k+1} \end{array}\right]=\left[\begin{array}{ccc} 1 & 0 & 0\\ 0 & 1-\frac{1}{R_{1}C_{1}} & 0\\ 0 & 0 & 1-\frac{1}{R_{2}C_{2}} \end{array}\right]\cdot \left[\begin{array}{c} {\hat{SOC}}_{k}\\ {\hat{U}}_{1,k}\\ {\hat{U}}_{2,k} \end{array}\right]+\left[\begin{array}{c} -\frac{\eta \;\cdot \;T}{Q_{N}}\\ \frac{1}{C_{1}}\\ \frac{1}{C_{2}} \end{array}\right]\cdot i_{k}+w_{k}$$

(14) $$U_{d,k}=U_{oc}\left({\hat{SOC}}_{k}\right)-{\hat{U}}_{1,k}-{\hat{U}}_{2,k}-i_{k}\cdot R_{0}+v_{k}$$

Linearizing the state-space equations shown in Equations (13) and (14), we obtain the local linear model of the lithium-ion battery as:

(15) $$x_{k+1}={\tilde{A}}_{k}x_{k}+B_{k}\cdot i_{k}+w_{k}$$

(16) $$y_{k}={\tilde{C}}_{k}x_{k}+D_{k}\cdot i_{k}+v_{k}$$

in which

(17) $${\tilde{A}}_{k}=\frac{\partial f\left(x_{k},u_{k}\right)}{\partial x_{k}}{|}_{x_{k}={\tilde{x}}_{k}}=\left[\begin{array}{ccc} 1 & 0 & 0\\ 0 & 1-\frac{1}{R_{1}C_{1}} & 0\\ 0 & 0 & 1-\frac{1}{R_{2}C_{2}} \end{array}\right]$$

(18) $$B_{k}={\left[-\frac{\eta \cdot T}{Q_{N}}\;\frac{1}{C_{1}}\;\frac{1}{C_{2}}\right]}^{T}$$

(19) $${\tilde{C}}_{k}=\frac{\partial f\left(x_{k},u_{k}\right)}{\partial x_{k}}{|}_{x_{k}={\tilde{x}}_{k}}=\left[\frac{\partial U_{oc}\left(SOC_{k}\right)}{\partial \mathrm{SOC}}{|}_{SOC={\tilde{SOC}}_{k}}-1-1\right]$$

(20) $$D_{k}=-R_{0}$$

Therefore, the approximation local linear time-varying model is estimated by KF to obtain the best estimation of SOC. More details about the algorithm can be seen in Refs. [25,26,27].

**Figure 10:**
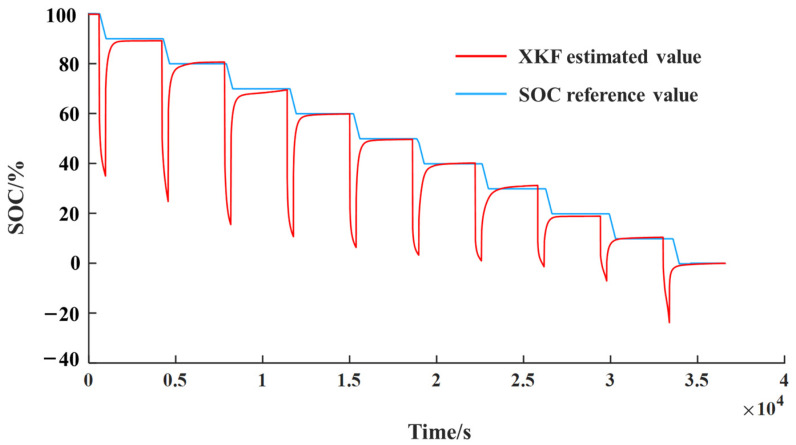
XKF estimated and reference values (experimental values) of SOC.

**Figure 11:**
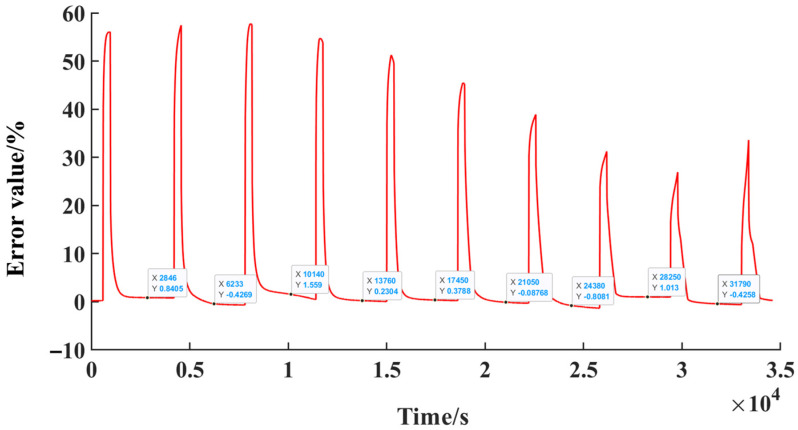
Relative errors between the XKF estimated and reference values (experimental values) of SOC.

Figure 10 shows the estimated XKF and reference values of SOC for the lithium-ion battery (experimental SOC values). The red line is the XKF estimation SOC values and the blue line is the SOC reference values, which are matched well, except for the points of inflection similar to the response voltage curve as shown in Figure 5b. The relative errors between the XKF estimated and reference values (experimental values) of SOC are shown in Figure 11. The relative errors are almost below 1%, except for the sudden changed in currents and voltages. Reasons for sudden change value are: XKF mainly predicts the battery SOC through the battery current and voltage; the battery current and voltage test results will not be sudden changes. Therefore, sudden changes will occur in the prediction results but will not influence the prediction results if they persevered convergences.

Therefore, it proves that the SOC estimation results using XKF are relatively stable and accurate. Moreover, compared to the EKF estimation results as shown in Figure 8, we found that EKF cannot effectively reduce the errors but continuously accumulate errors to be larger and larger.

## 5. Conclusions

To solve the stability problem that occurred in SOC estimation using the Kalman filter or extended Kalman filter, the eXogenous Kalman filter combined with the second-order RC equivalent circuit model for the lithium-ion battery is developed. The eXogenous Kalman filter is a cascade of auxiliary state estimators not dependent on the linear Kalman filter’s own state estimation but the design of the auxiliary state estimator as the alternate one. Therefore, the procedures are: (1) Construct the second-order RC equivalent circuit model of the lithium-ion battery and further determine the model parameters by experiments; (2) Build up the discretized state-space equations of the second-order RC equivalent circuit model to generate an auxiliary estimator; (3) Obtain the second-best estimation using auxiliary estimator; (4) Perform Kalman filter using approximation local linear time-varying model to obtain the best estimation of SOC. Experimental results show that SOC estimation using the eXogenous Kalman filter is superior to the commonly used extended Kalman filter for maintaining stability while having sufficient accuracy. It is worth pointing out that the influence of temperature and/or battery aging have not been directly considered in the present investigations and needs to be further investigated using the present method or its improved versions.

## Figures and Tables

**Table 1:** The relationship between *U_d_* and *SOC* during the test.

*SOC*/%	10	20	30	40	50	60	70	80	100
*U_d_*/V	3.3572	3.3510	3.5810	3.6492	3.7267	3.8182	3.9127	4.0082	4.1899

**Table 2:** Battery model parameter table.

$R_{0}$/Ω	$R_{1}$/Ω	$R_{2}$/Ω	$C_{1}/F$	$C_{2}$/F
0.04474	0.016603	0.0058259	10,358	18,862

## Data Availability

Not applicable.
